# Developmental Changes of Catecholamine-mediating Enzyme – Dopamine-β- Hydroxylase and Its Cofactors in Central and Peripheral Tissues and Serum of Long-Evans Rats

**Published:** 2012-09

**Authors:** M. Khalilur Rahman, M. Iqbal Choudhary, M. Rafiqul Islam, Rahman M. Hafizur

**Affiliations:** 1 *Department of Biochemistry and Molecular Biology, University of Dhaka, Dhaka 1000, Bangladesh;*; 2 *Dr. Panjwani Center for Molecular Medicine and Drug Research, International Center for Chemical and Biological Sciences, University of Karachi, Karachi 75270, Pakistan*

**Keywords:** developmental Changes, dopamine-β- hydroxylase, norepinephrine, adrenals, hypothalamus

## Abstract

Dopoamine-β-hydroxylase (DBH) is a catecholamine-synthesizing enzyme which catalyzes the formation of norepinephrine from dopamine. Fifty nine Long-Evans rats of 1 week-old were used to grow on normal diets in 7 different developmental stages, viz., 15 rats in 1 week-old group, 9 rats in 2 weeks-old group, and the remaining 35 rats were divided equally into five groups, 7 rats in each group of 5-, 8-, 12-, 15- and 27-weeks old for systematic developmental studies of DBH. At the end of each developmental period, weights of rats were recorded for that specific group and they were sacrificed. The brain tissues (caudate nucleus, hypothalamus, brain stem, colliculi, cerebral cortex and cerebellum) and peripheral tissues (liver, heart, kidney, adrenal, spleen, pancreas, lung and small intestine) and serum were collected. The brain tissues had the highest activity (expressed as nmole/min/g of wet weight tissue) of DBH at 5 weeks of age. The hypothalamus had the highest activities (11.5 ± 2.2) and the lowest activities were found in the cerebellum (5.7 ± 0.9). The peripheral tissues also showed the peak DBH activities at 5 weeks of age and adrenals had the highest activities (59.2 ± 7.0) among the central and peripheral tissues. The serum DBH activities were relatively low (1.3 ± 0.2) as compared to those in all other tissues. The highest serum DBH activities (1.28 ± 21 nmole/min/ml of serum) were also found in the 5- weeks-old rats. The specific activities of DBH were also measured in various developmental stages and the results were found to be in agreement with the DBH activities expressed in terms of gram of tissues or milliliter of serum. The K_m_ and V_max_ values for DBH were measured in the serum samples of each group and the highest V_max_ values (78.3 ± 21.2 pmol/min/mg protein) were obtained at 5 weeks of age; while the lowest K_m_ values (0.52 ± 0.04 mM) were obtained at this stage of age.

## INTRODUCTION

Dopamine-β-hydroxylase (DBH) [3,4-dihydroxyphenylethylamine, ascorbate: oxygen oxidoreductase (3-hydroxylating), EC 1.14.17.1; abbreviated DBH] is the enzyme responsible for the biosynthesis of catecholamine neurotransmitter, norepinephrine from dopamine, ultimately leading to the formation of epinephrine by the enzyme phenylethanolamine-N-methyl transferase in the mammalian tissues and serum (1, 2). The product norepinephrine (also called noradrenaline), is important biochemically and pharmacologically, because this monoamine is important intracellular messenger, such as neurotransmitter and hormone and involved in the regulation of neuronal functions, behavior and emotion of higher animals. DBH is a copper (Cu^2+^) and ascorbic acid (vitamin C) dependent enzyme and its activity is stimulated by the addition of dicarboxylic acids, such as fumaric acid (3).

DBH is an intraneuronal enzyme in the sympathetic neurons system and enzyme activity was found in the adrenal medulla (4), in the brain (5) and various sympathetically inervated organs in rats (6, 7). In humans and laboratory animals, DBH has been measured in plasma (8) and also in cerebrospinal fluid (CSF) (9). DBH being one of the key catecholamine-mediating enzymes has drawn much attention from clinical and pharmacological investigators as a possible index of the sympathetic nervous function. It is also linked to Parkinson’s disease (10, 11) and it was observed that a significant increase in DBH activity occurs in pheochromocytoma (12).

The developmental changes of rat serum aromatic L-amino acid decarboxylase (AADC) with L-3,4-dihydroxyphenylalanine (L-DOPA) and L-5-hydroxytryptophan (L-HTP) as substrates and of rat serum DBH have been studied at various stages of growth (13, 14) using the Wistar rats. In both of these enzymes studies, the peak activities showed at 3-weeks of age; but for AADC enzyme the peak activities again appeared at the adult age of Wistar rats. The studies have also been made on the influence of age, sex and hypoxia on plsma DBH activities in Wistar rats (15) and they found the maximum activities at the age of 5-, 30-, and 35-days of ages. In another studies (16) in an experimental model (juvenile Wistar rats), it was found that the highest activity appeared at the age of 5-weeks of age, then decreased till the age of 14-days and increased again in 14- to 35-days of age. The developmental changes of DBH activities have also been described in Sprague-Dawley rats by Behrens and Depocas (17) and Lamprecht and Wooten (18). The activities were very high at the early postnatal period, reaching the peak at about 16 days of age and approached adult activities at the age of around seven week. From these many studies, it is clear that the peak DBH activies are variable in various developmental stages of mammals; but no such studies have been made on DBH in the central- and peripheral-tissues and serum of rats at various age groups using the Long-Evans rats. This tempted us to do the systematic developmental studies of DBH enzyme activity and the concentrations of its cofactors (ascorbic acid and Cu^2+^) and related biochemical parameters, such as protein and glucose in the central (brain) tissues, such as, caudate nucleus, hypothalamus, brain stem, colliculi, cerebral cortex and cerebellum, and peripheral tissues (liver, adrenal, heart, kidney, spleen, pancreas, lung and small intestine) and serum of these rats at various age groups, namely, 1-, 2-, 5-, 8-, 12-, 15- and 27- weeks of age. The K_m_ and V_max_ values of serum DBH activity were also determined at various developmental age groups.

## MATERIALS AND METHODS

### Animals, Diets and Collection of Tissues and Blood

Fifty nine Long-Evan male rats of 1-week old were obtained from the animal house of International Centre for Diarrhoeal Diseases Research, Bangladesh. They were grouped as follows: 15 rats for 1-week old group, 9 rats for 2- weeks old group and 7 rats for each group of 5-, 8-, 12-, 15- and 27-weeks old. The weights of the rats of each group were recorded before decapitation. During the growth and development periods, the rats were fed the normal rat diets containing 30% proteins, 59% carbohydrate, 5% soybean oil, 5% salt mixture and 1% vitamin mixtures. The rats were maintained in clean cages with ad libitum access to water and food. The complete basal diets for rats were mixed with vitamin and salts and were kept at 4-8°C in closed containers in order to prevent oxidative decomposition of the labile ingredients, specially vitamins. The diets were prepared freshly, at an interval of 5-6 days. The fifteen rats of 1 week-old group were sacrificed by decapitation and their brains, peripheral tissues (liver, adrenals, heart, kidney, spleen, pancreas, lung and small intestine) and serum were collected. The serum was collected by keeping the blood in the cold temperature for about 3 hours and then centrifuged at 2500 rpm for 10 minutes and the top serum portion was collected. The tissues and serum were stored at -60°C until use. As the brains sizes and blood volumes were very small for 1- and 2- weeks old rats, we used the whole brains for biochemical analysis and the serum were pooled together to make 3 samples for each age group. We also collected the whole brains of each age group by decapapitaion of the additional rats for performing the experiments using the whole brains. For all other age groups, the brain tissues (caudate nucleus, hypothalamus, brain stem, colliculi, cerebral cortex and cerebellum) were collected separately. After each developmental periods the rats of that particular group were sacrificed and the central and peripheral tissues and serum were collected and stored at -60°C until use. The animals were maintained in the Animal House of Department of Biochemistry and Molecular Biology, University of Dhaka and were approved by the Animal Use and Care Committee of University of Dhaka, Dhaka, Bangladesh.

For the use in DBH enzyme assays and for other biochemical studies, the brain tissues were homogenized (5-10-fold dilution) with 0.32 M sucrose in a Potter glass homogenizer. The peripheral tissues were also homogenized in the same way, but with 0.25 M sucrose solution. The hypothalamus was diluted to 20 times due to small amounts of the tissue and because of its high activity of enzyme. The serum was not diluted for the assay purposes of DBH activity.

### The Assay of DBH Activity

The DBH activity was assayed by using the simple and rapid method of Kato *et al.* (19). For this assay Catalase, N-Ethylmaleimide, Octopamine, Ascorbic acid, Sodium Metaperiodate and Sodium Thiosulfate, DOWEX-50W-X4 (H^+^, 200-400 mesh) were obtained from Sigma Chem. Co. (St. Louis, Mo. 6317, USA); Tyramine. HCl and fumaric acid were from Calbiochem., SanDiego, Calif. 92112, USA; Pargyline. HCl was from Abbot Lab., North Chicago, III, 600064, USA The DOWEX-50W-X4 (H^+^, 200 – 400 mesh) was activated by cyclic washing with 1 N HCl and 1 M NaOH and finally it was equilibrated with 1 M Na-acetate buffer, pH 5.0 and stored in the same buffer. The assay method of DBH was simple, rapid and specific for measurement of nmol levels of the enzyme. DBH can also hydroxylate tyramine to octopamine, so tyramine is usually used as substrate for DBH instead of dopamine. The principle of DBH assay is based on the enzymatic conversion of tyramine to octopamine. The same volume of boiled enzyme was used as blank and 2.0 nmoles of octopamine was used in another blank incubation mixture as internal standard. The incubation temperature and time were 37°C and 30 minutes, respectively. The product octopamine was isolated by using small column containing 0.2 ml of activated DOWEX-50W- X4 (H^+^, 200-400 mesh) resin. The column was washed with water and then the adsorbed amines were eluted with 1.0 ml of 3 N NH_4_OH solution. The octopamine in the eluate was converted to p-hydroxy-benzyldehyde by adding 10 µl of 2% NaIO_4_ solution. The excess NaIO_4_ was reduced by adding 10 µl of 10% Na_2_S_2_O_5_ solution. The solution was then acidified by adding 0.5 ml of 6 N HCl. The p-hydroxy-benzyldehyde in the solution was extracted with 5.0 ml of ethyl ether. The ether phase was again extracted with 1.0 ml of 3 N NH_4_OH for the measurement of the absorbance at 330 nm using the experimental, blank and internal standard samples. The absolute amount of octopamine formed was calculated using the following:

$$\text{Octopamine}\;\text{formed}\;(\text{nmoles})\;=\;\frac{\left(\text{Absorbance}\;\text{of}\;\text{experimental})-(\text{Absorbance}\;\text{of}\;\text{blank}\right)}{\left(\text{Absorbance}\;\text{of}\;\text{internal}\;\text{standard})-(\text{Absorbance}\;\text{of}\;\text{blank}\right)}\;\times \;2.0$$

The endogenous inhibitors that interfere with the assay of DBH in vitro were inactivated by adding excess N-methylmaleimide.

### Detrmination of K_m_ and V_max_ Values of Rat Serum DBH

DBH activity in different substrate concentrations was measured and the velocities of the reactions were determined for each substrate concentration. Then the values for (1/v) were plotted against those of the (1/S)’s to obtained the Lineweaver-Burk plot (20). The K_m_ and V_max_ for BDH of rat serum of different developmental age groups were measured to compare the kinetic relationships of DBH with each other groups. The concentrations of Cu^2+^ in the serum and tissues was determined by the procedure using an Atomic Absorption Spectrophotometer (AAS, Pye-Unicam SP9). The estimation of ascorbic acid in the serum and various tissues of rats were done by the colorimetric ascorbic acid assay kit (BioVision Inc. CA, USA) immediately after collection of the samples. The estimation of total protein in the serum and various tissues of rats were determined with the protein assay reagent (Bio-Rad Laboratories, CA, USA) using bovine serum albumin as standard. The serum glucose was measured by glucose oxidase method (Randox Laboratories, Antrim, UK).

### Statistical Analysis

All the data were analyzed using the Statistical Package for Social Sciences (SPSS) (version 11.0 for Windows, SPSS Inc., Chicago, USA). Data were expressed as mean and standard deviation ( ± SD). Student t-test (two-tailed) was used to evaluate statistical differences between the two study groups. A P-value of ≤0.05 was the criterion for a statistically significant difference.

## RESULTS

### DBH Activities in Central (Brain) and Peripheral Tissues and in Serum of Long-Evans Rats at Various Developmental Stages

**Figure 1:**
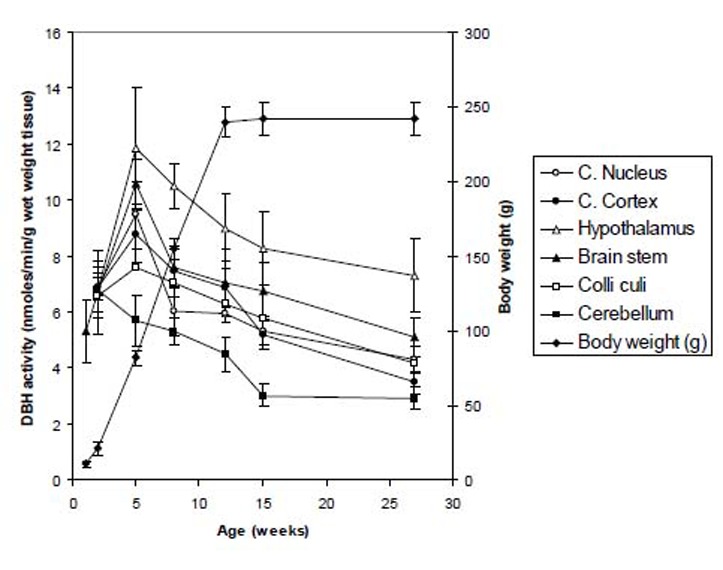
Developmental changes of DBH in brain tissues of rat with body weight. The DBH activities at 1- week and 2- weeks old rats were the values (5.1 ± 1.1 and 6.99 ± 1.5, respectively) of the whole brains as it was difficult to separate the various brain tissues. The activities of DBH increased sharply from 1- week to 2- weeks of age and then to the peak activities in all the brain tissues at the age of 5 weeks. The hypothalamus had the highest activity of 11.85 ± 2.2, followed by brain stem (10.6 ± 0.9), caudate nucleus (9.48 ± 1.2), cerebral cortex (8.8 ± 1.2), colliculi (7.58 ± 0.5) and cerebellum (5.68 ± 0.9). After 5 weeks of age the activities again gradually decreased to significantly low values (*p*<0.05) at the adult stages of 8- and 15- weeks of age as compared to the adult stages of rats. However, in the old age of 27- weeks of age, the values decreased more to the values of 7.32 ± 1.3 (hypothalamus), 5.1 ± 0.7 (brain stem), 4.29 ± 0.5 (caudate nucleus), 4.2 ± 0.8 (colliculi), 3.5 ± 0.4 (cerebral cortex ) and 2.93 ± 0.4 (cerebellum). Data are mean ± SD (n=7).

**Figure 2:**
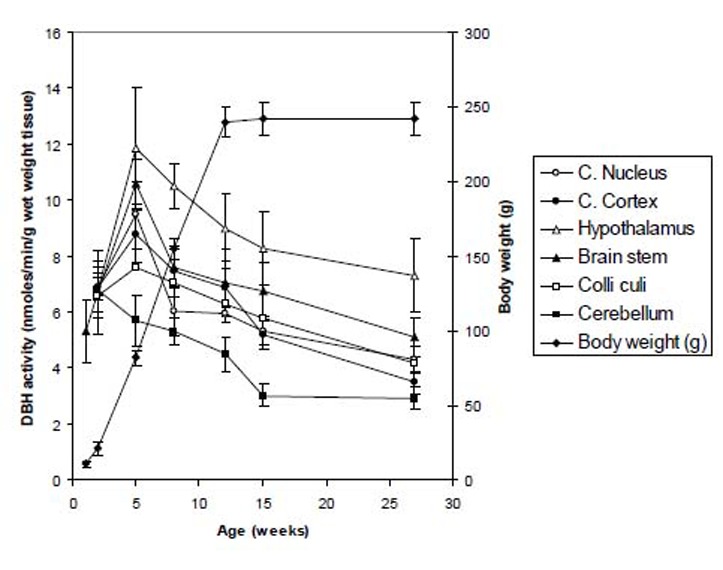
Developmental changes of DBH in peripheral tissues and serum of Long-Evans rats. The peripheral tissues and serum also showed the highest DBH activities at 5- weeks of age in the same rats as described in Figure 1. The adrenal gland showed the highest activity (59.25 ± 7.0) at the age of 5- weeks, followed by 8- weeks (51.04 ± 6.4), 2- weeks (45.27 ± 5.0), 12- weeks (39.12 ± 3.2), 15- weeks (33.1 ± 3.5), 27- weeks (29.03 ± 2.4) and 1- week (26.3 ± 3.5) of age. The sharp rise in the activity of DBH was found in pancreas in 2- weeks of age (14.82 ± 1.6), the values are near the activity (15.17 ± 1.8) at 5- weeks of age. The peak DBH activities were also found at 5- weeks of age for other peripheral tissues, followed by spleen, liver, small intestine, kidney and lung. The other age groups they are relatively low as compared to 5- weeks of age. The values in the serum were low in all the developmental stages, they were 0.55 ± 0.06, 0.85 ± 0.08, 1.28 ± 0.21, 0.73 ± 0.13, 0.49 ± 0.07, 0.41 ± 0.12 and 0.31 ± 0.08, for 1-, 2-, 5-, 8-, 12-, 15- and 27- weeks of age, respectively. The values were variable in all the peripheral tissues and serum, but the peak activities appeared at the 5- weeks of age of rats. The values were generally low in the old age (27- weeks old) (*p*<0.05) as compared to that in the 5- weeks (peak values) and 15- weeks (adult stage) of age of rats. Data are mean ± SD (n=7).

DBH activities in different parts of the brain (cerebral cortex, cerebellum, brain stem, caudate nucleus, colliculi and hypothalamus) and in various peripheral tissues (heart, lung, kidney, liver, spleen, small intestine, adrenal and pancreas) and in serum of rats at various stages of development, namely, 1-, 2-, 5-, 8-, 12-, 15- and 27-weeks of age, were measured by a very sensitive method(approximately 1 nmoles) as described in the Materials and Methods. The results are presented in Figures 1 and 2. In these studies, one unit of DBH activity is expressed as nmole/min/g wet wt. tissue or ml of serum. Figure 1, shows the developmental changes of DBH in brain tissues of Long-Evans rats with body weight (g). The DBH activities at 1- week and 2- weeks old rats were 5.1 ± 1.1 and 6.99 ± 1.5 units, respectively, using the whole brains as the brains were very small and it was difficult to separate the various brain tissues at these age groups. The activities of DBH increased sharply from 1- week to 2- weeks of age and then to the peak activities in all the brain tissues at the age of 5 weeks. The hypothalamus had the highest activity of 11.85 ± 2.2, followed by brain stem (10.6 ± 0.9), caudate nucleus (9.48 ± 1.2), cerebral cortex (8.8 ± 1.2), colliculi (7.58 ± 0.5) and cerebellum (5.68 ± 0.9). After 5 weeks of age the activities gradually decreased to significantly low values (*p*<0.05) at the stages of 8-, 12- and 15- weeks of age. The age group of 15- weeks is considered as the fully adult stages of rats. However, in the old age of 27-weeks of age, the values decreased further more to the values of 7.32 ± 1.3 (hypothalamus), 5.1 ± 0.7 (brain stem), 4.29 ± 0.5 (caudate nucleus), 4.2 ± 0.8 (colliculi), 3.5 ± 0.4 (cerebral cortex ) and 2.93 ± 0.4 (cerebellum).

Developmental changes of DBH in peripheral tissues and serum of Long-Evans rats are shown in Figure 2. The peripheral tissues and serum also showed the highest DBH activities at 5- weeks of age in the same rats as described in Figure 1. The adrenal gland showed the highest activity (59.25 ± 7.0) at the age of 5- weeks, followed by 8- weeks (51.04 ± 6.4), 2-weeks (45.27 ± 5.0), 12- weeks (39.12 ± 3.2), 15- weeks (33.1 ± 3.5), 27- weeks (29.03 ± 2.4) and 1-week (26.3 ± 3.5) of age. The sharp rise in the activity of DBH was found in pancreas in 2- weeks of age (14.82 ± 1.6), the values are near the activity (15.17 ± 1.8) at 5- weeks of age. The peak DBH activities were also found at 5- weeks of age for other peripheral tissues, followed by spleen, liver, small intestine, kidney and lung. The other age groups the values are relatively low as compared to those at 5- weeks of age. The values in the serum were very low in all the developmental stages, namely, 0.55 ± 0.06, 0.85 ± 0.08, 1.28 ± 0.21, 0.73 ± 0.13, 0.49 ± 0.07, 0.41 ± 0.12 and 0.31 ± 0.08, for 1-, 2-, 5-, 8-, 12-, 15- and 27-weeks of age, respectively. The values were variable in all the peripheral tissues and serum, but the peak activities appeared at the 5- weeks of age of rats. The values were generally low in the old age (27-weeks old) (*p*<0.05) as compared to that in the 5-weeks (peak values) and 15-weeks (adult stage) of age of rats.

The specific activities of DBH were measured in the serum, central and peripheral tissues at various stages of Long-Evans rats as shown in Table 1. The data of serum, liver, lung, heart, small intestine, caudate nucleus, hypothalamus and brain stem were available for analysis. All these results showed the peak DBH specific activities at 5- weeks of age; some values at 2-weeks of age were close but lower to those at 5- weeks of age. The brain tissues, namely, caudate nucleus, hypothalamus and brain stem showed the highest specific activities of 130.0 ± 16.4, 102.9 ± 19.1 and 124.2 ± 14.0, respectively, at the age of 5- weeks. The peripheral tissues, like, small intestine, liver and heart had the high specific activies of DBH with the lowest values of that in lung tissue. But all of these tissues had the peak DBH specific activities at the 5- weeks of age. The DBH specific activities in serum was relatively low in all the developmental stages, but the peak activities (1.3 ± 0.2) were obtained at the age of 5 weeks.

**Table 1:** Specific activities of DBH in the serum, central and peripheral tissues of Long-Evan rats in different developmental stages^a^

Name of the tissues	Specific activities of DBH at different developmental stages (weeks) (pmol/min/mg protein)						
	1 (n=7)	2 (n=7)	5 (n=7)	8 (n=7)	12 (n=7)	15 (n=7)	27 (n=7)

Serum	27.3 ± 2.9	28.40 ± 2.6	30.2 ± 4.9	18.8 ± 3.3	11.2 ± 1.6	8.7 ± 2.5	8.7 ± 2.2
Liver	40.1 ± 5.8	93.4 ± 12.2	131.6 ± 14.9	87.5 ± 9.7	80.7 ± 10.9	59.6 ± 8.4	72.6 ± 10.2
Lung	77.9 ± 13.4	81.4 ± 10.1	88.0 ± 8.2	67.0 ± 8.9	49.2 ± 6.9	34.8 ± 1.9	19.8 ± 0.7
Heart	120.9 ± 21.9	131 ± 20.9	140.9 ± 14.7	79.4 ± 6.9	55.8 ± 7.1	57.5 ± 5.5	33.6 ± 14.2
S. intestine	66.0 ± 8.3	115 ± 16.0	157.7 ± 18.8	114 ± 15.0	72.0 ± 9.2	50.0 ± 5.6	50.4 ± 5.0
C. nucleus	89.9 ± 16.0^b^	85.2 ± 3.0^b^	130.0 ± 16.4	60.8 ± 9.0	61.1 ± 3.0	54.9 ± 5.1	30.2 ± 3.4
Hypoth thalamus	89.9 ± 16.0^b^	85.2 ± 3.0^b^	102.9 ± 19.1	77.6 ± 5.9	65.0 ± 8.6	58.6 ± 9.2	56.6 ± 10.0
Brain stem	89.9 ± 16.0^b^	85.2 ± 3.0^b^	124.2 ± 14.0	69.3 ± 6.4	59.4 ± 10.1	54.0 ± 8.0	48.1 ± 6.6

a Data are presented as mean ± SD;

b Whole brains were used as the individual tissues were too small at these stages.

### K_m_ and V_max_ Values of the Healthy Rat Serum DBH at Different Developmental Age Groups

The kinetics of DBH in serum of the chosen age groups were measured by incubating the enzyme with different concentrations of tyramine as substrate. The rate of conversion of substrate into product was plotted on a Lineweaver-Burk plot and from that graph the V_max_ (the highest velocity of enzyme activity) and the K_m_ (substrate concentrations at which the enzyme activity is the half of the V_max_) values were calculated as shown in Table 2. The highest V_max_ values (78.3 ± 21.2 pmol/min/mg of protein)) were found at 5 weeks of age, the K_m_ values were lowest (0.52 ± 0.04 mM) at this age group. The Vmaz and K_m_ values were 75.0 ± 20.0 and 0.79 ± 0.2; 66.3 ± 24.8 and 1.22 ± 0.5; 53.7 ± 10.3 and 0.92 ± 0.05 for 2-, 1- and 8- weeks of age respectively. The values were almost similar at the adult levels of 12- and 15- weeks of age. At the 27- weeks of age the proteins concentrations were low, so the V_max_ values were found to be elevated little (44.0 ± 14.0).

**Table 2:** K_m_ and V_max_ values of the healthy rat serum DBH at different developmental age groups^a^

Age of the rats (weeks)	K_m_ (mM) (n=7)	V_max_ (pmol/min/mg protein) (n=7)

1 week	1.22 ± 0.5	66.3 ± 24.8
2 week	0.79 ± 0.2	75.0 ± 20.0
5 week	0.52 ± 0.04^b^	78.3 ± 21.2^c^
8 week	0.92 ± 0.05	53.7 ± 10.3
12 week	1.37 ± 0.6	38.1 ± 10.4
15 weeks (adult rats)	1.60 ± 0.1^b^	37.8 ± 7.9^c^
27 weeks (old rats)	2.10 ± 0.4	44.0 ± 14.0

a Data are presented as Mean ± SD;

b *P*<0.05 versus highest and lowest K_m_ values;

c *P*<0.05 versus highest and lowest V_max_ values.

### Protein Contents of Tissues and Serum at Various Developmental Stages of Rats

The total protein contents were measured in various brain tissues (cerebral cortex, cerebellum, brain stem, caudate nucleus, colliculi and hypothalamus) as well as in various peripheral tissues, namely, heart, liver, lung, spleen, kidney, adrenal, small intestine and pancreas and serum of seven developmental stages (1 week- , 2 weeks-, 5- weeks-, 8 weeks-, 12 weeks-, 15 weeks-and 27 weeks-old) of Long-Evans rats as shown in Tables 3 and 4.

**Table 3:** Protein contents of the central tissues of various developmental stages of rats^a^

Name of the tissues	Protein contents at different age groups (in weeks) of rats (mg/g wet weight tissue)						
	1 (n=7)	2 (n=7)	5 (n=7)	8 (n=7)	12 (n=7)	15 (n=7)	27 (n=7)

C. nucleus	-	-	73.0 ± 0.9^c^	99.2 ± 8d	97.3 ± 4.2^d^	97.1 ± 15^d^	95.2 ± 11^d^
Cerebral cortex	-	-	126.4 ± 3^c^	148.2 ± 3^d^	149.7 ± 5^d^	152 ± 7.1^d^	116 ± 3.4^c^
Hypoth thalamus	-	-	115.2 ± 3^c^	135.3 ± 9^d^	138.5 ± 11^d^	141 ± 13.9^d^	129.3 ± 3.4^c^
Brain stem	65.5 ± 8.3b,^c^	78.6 ± 12b,^c^	85.3 ± 3.1^c^	109.3 ± 3^c^	118.3 ± 14	125 ± 3.9^d^	106 ± 5.1^c^
Colliculi	-	-	106.2 ± 4^c^	121 ± 14^d^	120.5 ± 3.8^d^	123.6 ± 6^d^	102.4 ± 3.2^c^
Cere-bellum	-	-	112.4 ± 4^c^	132.1 ± 8^d^	141.4 ± 4.3^d^	150 ± 11^d^	122 ± 3.5^c^

a Data are presented as mean ± SD;

b Whole brains were used as the individual tissues were too small at these stages;

c *P*<0.05 values versus high values;

d Value of protein contents in tissues.

**Table 4:** The protein content in peripheral tissues and serum of rats at different developmental stages^a^

Name of the Tissues	Protein content at different age groups (in weeks) of rats (mg/g wet weight tissue)						
	1 (n=7)	2 (n=7)	5 (n=7)	8 (n=7)	12 (n=7)	15 (n=7)	27 (n=7)

Kidney	68 ± 4.3^c^	84.8 ± 6	112.8 ± 11	136.4 ± 5^d^	143 ± 3.2^d^	149 ± 8.5^d^	145 ± 4.8^d^
Liver	179.2 ± 15^d^	122 ± 3.9	114.0 ± 0.9	103.0 ± 9.0	91.6 ± 3.7^c^	106.5 ± 2^c^	73.2 ± 2^c^
Lung	74.4 ± 5.3^c^	88.8 ± 3.2^c^	97.2 ± 1.9^c^	106.0 ± 12^c^	130 ± 15^d^	140.8 ± 3.1^d^	116 ± 4.2^c^
Spleen	71.3 ± 6.8^c^	87.0 ± 1.9^c^	161.0 ± 3.8^d^	173.2 ± 2^d^	169 ± 1.3^d^	167.2 ± 4^d^	140 ± 9.8^d^
Heart	68.4 ± 4.3^c^	86 ± 7.3	95.0 ± 4.9	115.2 ± 7^d^	101.3 ± 1^d^	89.6 ± 7	95.2 ± 1.0
S. intestine	108.4 ± 1^d^	87 ± 9	90.0 ± 4.3	80.0 ± 1.3^c^	107.0 ± 4.5	106 ± 3.2^d^	93.2 ± 8.1
Pancreas	56.2 ± 1.9^c^	72.4 ± 3.1	88.0 ± 6.9	99.6 ± 1.5^d^	106 ± 10d	109 ± 3.2^d^	95 ± 1.8
Serum^b^	30.1 ± 4.3^c^	29.92 ± 1	42.4 ± 8.5^d^	38.7 ± 1	43.5 ± 3.8^d^	47 ± 4.5^d^	35.5 ± 1.3^c^

a Data were presented as mean ± SD;

b Protein contents were expressed as mg/ml of serum;

c *P*<0.05 versus high protein contents;

d Contents of tissues and serum.

The protein contents (mg/g wet wt of the tissues) of whole brains of rats of 1 and 2 weeks old were low (65.5 ± 8.3 and 78.6 ± 12.0, respectively) as compared to those of other age groups. Similarly, the protein contents of all the brain tissues of 5 weeks old rats were significantly lower (i.e., in brain stem, the values are 85.5 ± 3.1) than those of 12 and 15 weeks old rats (118.3 ± 14.0 and 125.0 ± 3.9, respectively) which were considered as the adult rats. The protein contents of caudate nucleus of 5 weeks old rats were even lower than that of 2 weeks old rats.

The contents of protein are variable in the various peripheral tissues, but the lowest amount was in the tissues of 1 week old rats with the exceptions in the small intestine and liver which had the activities of 108.4 ± 1 and 179.2 ± 15 mg/g wet weight of tissues, respectively, as shown in Table 4. The contents in the liver were decreased gradually to the old age (27 weeks), but for the tissues, like, kidney,, lung, spleen, pancreas and small intestine, the highest contents of proteins were in the adult age groups (12 and 15 weeks). The protein contents in all of the tissues were lowered in the old age (27 weeks), except the kidney values (145.0 ± 4.8) as compared to adult age groups. The protein contents in the serum at various developmental age groups were very low as compared to those in the brain- and peripheral- tissues as the protein is used in many others purposes in the tissues. Although DBH had the peak activities in all the brains and peripheral tissues and serum at 5 weeks of age, the protein contents did not follow the same patterns as shown in Tables 3 and 4.

### Ascorbic Acid Content at Various Developmental Stages of Rats

Ascorbic acid concentrations (mg/g wet weight of tissue) in the central and peripheral tissues and serum of rats were measured immediately after the collection of the tissues and serum as it is easily destroyed by long storage. The highest amount was found in all the developmental stages of the adrenal glands of rats (0.90 ± 0.01 in 1- week old rats to 4.40 ± 0.40 in 27- weeks old rats) as compared to other central and peripheral tissues and serum of rats. The results are shown in Table 5. The concentrations were lowest in the tissues of 1 and 2 weeks of age with the exception of spleen, adrenal and hypothalamus. Liver had the highest concentrations of ascorbic acids at 5- and 8- weeks of age; the values are 0.24 ± 0.02 and 0.24 ± 0.03, respectively. The high concentrations of ascorbic acid were found in the developmental stages of other age groups. The contents became low in many of the tissues at the old age with the exception of adrenal glands. The peak concentrations of ascorbic acid were observed at 5- weeks of age in kidney, lung, pancreas, hypothalamus and serum.

**Table 5:** The ascorbic acid (Vit-C) content in different peripheral and central tissues and serum of rats at different developmental age groups^a^

Name of the Tissue	Ascorbic acid content at different ages (in weeks) of rats.(mg/g wet weight tissue)						
	1 (n=7)	2 (n=7)	5 (n=7)	8 (n=7)	12 (n=7)	15 (n=7)	27 (n=7)

Kidney	0.05 ± 0.02^c^	0.11 ± 0.03	0.17 ± 0.04	0.14 ± 0.18	0.10 ± 0.03	0.10 ± 0.1^c^	0.09 ± 0.02^c^
Liver	0.10 ± 0.01^c^	0.15 ± 0.04	0.24 ± 0.02^d^	0.24 ± 0.03	0.21 ± 0.04	0.22 ± 0.03	0.23 ± 0.04
Lung	0.15 ± 0.03^c^	0.21 ± 0.03	0.29 ± 0.05^d^	0.28 ± 0.04^d^	0.26 ± 0.02	0.23 ± 0.04	0.20 ± 0.02^c^
Spleen	0.20 ± 0.03^c^	0.26 ± 0.06^d^	0.34 ± 0.07^d^	0.31 ± 0.07	0.35 ± 0.04^d^	0.37 ± 0.1^d^	0.42 ± 0.1^d^
Adrenal	0.90 ± 0.01^d^	0.95 ± 0.05^d^	1.62 ± 0.09^d^	1.85 ± 0.06^d^	2.30 ± 0.6^d^	3.4 ± 0.7^d^	4.46 ± 0.4^d^
Heart	0.03 ± 0.01^c^	0.07 ± 0.01	0.11 ± 0.02	0.12 ± 0.06	0.14 ± 0.05	0.13 ± 0.03	0.10 ± 0.02
S.Intestine	0.15 ± 0.02^c^	0.23 ± 0.08	0.33 ± 0.06^d^	0.3 ± 0.04^d^	0.33 ± 0.1^d^	0.39 ± 0.04^d^	0.34 ± 0.05^d^
Pancreas	0.03 ± 0.01^c^	0.09 ± 0.01^c^	0.13 ± 0.01	0.11 ± 0.04	0.09 ± 0.02^c^	0.1 ± 0.01^c^	0.07 ± 0.01^c^
Hypothala	0.21 ± 0.04^d^	0.26 ± 0.05^d^	0.30 ± 0.04^d^	0.28 ± 0.02^d^	0.28 ± 0.02^d^	0.27 ± 0.1^d^	0.26 ± 0.03^d^
Serum^b^	0.41 ± 00.1^d^	0.65 ± 0.1^d^	0.86 ± 0.1^d^	0.81 ± 0.1^d^	0.79 ± 0.1^d^	0.75 ± 0.01^d^	0.64 ± 0.01^d^

a Data are presented as mea ± SD;

b Ascorbic acid concentration is expressed as mg/ml of serum;

c *P*<0.05 versus high values;

d Value of ascorbic acid concentrations in the tissues and serum.

### Copper Content at Various Developmental Stages of Rats

The concentrations (ppm) of copper are widely variables in the various central and peripheral tissues and serum of rats as shown in Table 6. The contents of Cu^2+^ in the liver, kidney, heart and hypothalamus (brain tissue) are high as compared to other tissues. The concentrations remained high even in the old age (kidney, liver, spleen and heart). The lowest values (1.32 ± 0.20) were found in lung of 1- week old rat as compared to the highest values in liver (38.0 ± 4.50) of 27- weeks old rats. There is no specific peak values at 5- weeks old rats. The concentrations in the serum were low (the highest serum values were 0.52 ± 0.00 in 8- weeks old rats and the lowest values of 0.32 ± 0.00)as compared to central and peripheral tissues.

**Table 6:** The copper content of rat serum in central and peripheral tissues at different developmental stages^a^

Name of the Tissue	Amount of copper (ppm) at different developmental stages (in weeks)						
	1 (n=7)	2 (n=7)	5 (n=7)	8 (n=7)	12 (n=7)	15 (n=7)	27 (n=7)

Kidney	3.90 ± 0.3^b^	6.3 ± 0.7^c^	8.1 ± 1.3^c^	11.8 ± 1.4^c^	11.4 ± 2.1^c^	14.3 ± 1.8^c^	23.6 ± 3.6^c^
Liver	5.20 ± 0.9^b^	15.9 ± 1.9^c^	22.0 ± 4.3^c^	24.7 ± 3.5^c^	25.6 ± 3.9^c^	29.3 ± 6.1^c^	38.0 ± 4.5^c^
Lung	1.32 ± 0.2^b^	3.20 ± 0.6^b^	3.90 ± 0.3	4.80 ± 0.1	5.30 ± 0.7^c^	5.20 ± 0.4^c^	4.40 ± 0.3^b^
Spleen	2.10 ± 0.5^b^	4.10 ± 0.3^b^	8.50 ± 1.3^c^	9.30 ± 0.8^c^	11.7 ± 0.9^c^	15.0 ± 2.1^c^	21.1 ± 4.1^c^
Adrenal	2.19 ± 0.2^b^	3.25 ± 0.4^c^	7.19 ± 1.3^c^	8.30 ± 0.9^c^	1.32 ± 0.8^c^	6.9 ± 1.2^c^	5.83 ± 0.9
Heart	5.00 ± 0.5^b^	8.10 ± 0.9^c^	15.5 ± 2.3^c^	21.8 ± 0.9^c^	19.4 ± 2.5^c^	18.5 ± 0.9^c^	35.0 ± 3.8^c^
S. intestine	3.90 ± 0.3^b^	8.30 ± 1.1^c^	10.5 ± 1.3^c^	16.7 ± 1.6^c^	15.5 ± 3.2^c^	11.3 ± 1.2^c^	8.61 ± 1.2^c^
Pancreas	2.51 ± 0.4^b^	4.35 ± 0.9^b^	6.01 ± 0.3^c^	6.30 ± 0.4^c^	8.65 ± 0.8^c^	8.05 ± 1.3^c^	6.08 ± 0.8^c^
Hypothalamus	8.30 ± 0.7^c^	10.2 ± 1.0^c^	12.1 ± 1.2^c^	13.5 ± 1.2^c^	11.3 ± 1.3^c^	12.5 ± 0.9^c^	9.20 ± 1.2^c^
Serum	0.32 ± 0.0^d^	0.44 ± 0.0^e^	0.46 ± 0.1^e^	0.52 ± 0.0^e^	0.47 ± 0.1^e^	0.59 ± 0.1^e^	0.33 ± 0.0^d^

a Data are presented as mean ± SD;

b *P*<0.05 versus the high values;

c Value of copper at different developmental stages of rats;

d *P*<0.05 versus the high values;

e Value of copper in serum of rats at different developmental stages.

The contents of glucose in the whole brains, peripheral tissues and serum of rats at various developmental stages were significantly different from each other as stated below: The values (n=7) were expressed as mg/g wet weight tissue (for serum, mg/dl of serum). For serum, they are 42.2 ± 0.3, 92.8 ± 1.6, 96.4 ± 7.0, 102.4 ± 17.0, 105.7 ± 13.0, 76.3 ± 9.4 and 96 ± 14.3, for 1-, 2-, 5-, 8-, 12-, 15 and 27- weeks old rats, respectively. Similarly, the values for whole brains, adrenals and liver were 0.7 ± 0.1, 0.9 ± 0.2, 1.5 ± 0.1, 1.6 ± 0.2, 1.8 ± 0.2, 1.9 ± 0.4, 2.0 ± 0.1;2.1 ± 0.2, 4.6 ± 0.7, 5.1 ± 0.8, 6.1 ± 0.9, 6.4 ± 0.6, 6.8 ± 0.5, 6.7 ± 0.9 and 14.2 ± 1.6, 16.0 ± 1.7, 16.6 ± 1.3, 17.5 ± 1.4, 17.4 ± 0.6, 19.5 ± 0.8, 21.2 ± 2.1, respectively. In the serum, the highest concentrations of glucose were 105.7 ± 13.0 at 15- weeks of age (adult age), followed by 102.4 ± 17.0 (12- weeks of age), 96.4 ± 7.0 (5- weeks of age) and 96.0 ± 14.3 (old age, 27- weeks of age). Although the peak activities of glucose appeared in different developmental stages of rat serum, brains and peripheral tissues, due to various metabolic and functional roles of glucose; it also plays very significant roles with DBH regulation as it is a glycoprotein in nature.

## DISCUSSION

The sympathetic and parasympathetic nerve endings secrete one of the two synaptic transmitter substances, norepinephrine or acetylcholine. Norepinephrine discharges many biological effects to the tissues and blood. For example, it accelerates the heart beat, raises the blood pressure, constricts the blood vessels of the skin, prepares the body with stressful and elevates blood glucose, etc.

DBH is an important enzyme in the synthesis of norepinephrine and epinephrine. It is suggested that DBH- catalyzed reaction step is the rate limiting step in the formation of both norepinephrine and epinephrine (1, 6). DBH being one of the key catecholamine-mediating enzymes has drawn much attention from clinical and pharmacological investigators as a possible index of the sympathetic nervous function. It is also linked to Parkinson’s disease (10, 11) and it was observed that a significant increase in DBH activity occurs in pheochromocytoma (12). A lot of other studies have also been done on this enzyme based on its various biochemical clinical and pharmacological importance and properties (21-23).

Up to now several developmental studies have been done on the neurochemical enzymes of mammals. The developmental changes of rat serum aromatic L-amino acid decarboxylase (AADC) with L-3,4-dihydroxyphenylalanine (L-DOPA) and L-5-hydroxytryptophan (L-HTP) as substrates and of rat serum DBH have been studied at various stages of growth (13, 14) using the Wistar rats. In both of these enzymes studies, the peak activities showed at 3- weeks of age; but for AADC enzyme the peak activities again appeared at the adult age of Wistar rats. The studies have also been made on the influence of age, sex and hypoxia on plsma DBH activities in Wistar rats (15) and they found the maximum activities at the age of 5-, 30-, and 35-days of ages. In another studies (16) in an experimental model (juvenile Wistar rats), it was found that the highest activity appeared at the age of 5- weeks of age, then decreased till the age of 14-days and increased again in 14- to 35-days of age. The developmental changes of DBH activities have also been described in Sprague-Dawley rats by Behrens and Depocas (17) and Lamprecht and Wooten (18). The activities were very high at the early postnatal period, reaching the peak at about 16 days of age and approached adult activities at the age of around seven week. From these many studies, it is clear that the peak DBH activies are variable in various developmental stages of mammals. Up to now no systematic developmental studies of DBH enzyme hasve been done on Long-Evans rats. Therefore, we undertook the present systematic study of the tissue distribution of DBH activities in 8 different peripheral tissues and 6 different parts of the brain and in the serum of Long-Evans rats at 7 developmental stages. It was not possible to separate the different parts of the brain at the age of 1 week or 2 weeks. So, we measured the DBH activity by taking the whole brains at these two developing groups. At the age of 1 week the activity was low but the activity increased by a value of 30% during the age of two weeks. This might be due to higher sympathetic nerve activity. The highest activity was found in the group of 5 weeks aged rats and then the activity was started to decrease moderately up to the age of 12 weeks. After then, the value was further decreased at slow rate up-to the old age of rats. It was observed that the activities of DBH increased sharply from 1- week to 2- weeks of age and then to the peak activities in all the brain tissues at the age of 5 weeks. The hypothalamus had the highest activity, followed by brain stem, caudate nucleus, cerebral cortex, colliculi and cerebellum. After 5 weeks of age the activities gradually decreased to significantly low values at the stages of 8-, 12- and 15- weeks of age. The age group of 15- weeks is considered as the fully adult stages of rats. However, in the old age of 27- weeks of age, the values decreased further more in hypothalamus, brain stem, caudate nucleus, colliculi, cerebral cortex and cerebellum. The hypothalamus is regarded as a higher nervous center for the control of lower autonomic centers in the brain stem and spinal cord. The lower DBH activity was found in cerebral cortex, colliculi and cerebellum.

The peripheral tissues and serum also showed the highest DBH activities at 5- weeks of age in the same rats. The adrenal gland showed the highest activity at the age of 5- weeks, followed by 8- weeks, 2- weeks, 12- weeks, 15- weeks, 27- weeks and 1- week of age. The sharp rise in the activity of DBH was found in pancreas in 2- weeks of age, the values are near the activity at 5- weeks of age. The peak DBH activities were also found at 5- weeks of age for other peripheral tissues, followed by spleen, liver, small intestine, kidney and lung. The other age groups the values are relatively low as compared to those at 5- weeks of age. The values in the serum were very low in all the developmental stages of 1-, 2-, 5-, 8-, 12-, 15- and 27- weeks of age. However, the values were variable in all the peripheral tissues and serum, but the peak activities appeared at the 5- weeks of age of rats. The values were generally low in the old age of Long-Evans rats.

Among the sympathetically innervated peripheral tissues, rat adrenal gland had the highest DBH activity. An increase of 72% of DBH activity was found in 2 weeks aged rats than that of 1 week old rats. The peak value was obtained at the age of 5 week; the values gradually decreased up to the old age 27 weeks. The DBH activities in the most other peripheral tissues followed the same ascending and descending patterns but their values and ranges were different.

In serum, DBH activity increased sharply up to 5 weeks of age, then it started to decline gradually as the age proceeded. The activities were in agreements with the K_m_ and V_max_ values of DBH at these developmental stages. DBH is a glycoprotein in nature and K_m_ and V_max_ values of DBH showed more or less in agreement results in the tissues and serum from the birth to the adult age. The specific activities of DBH and its V_max_ and K_m_ values were measured and the results were explainable and were in agreements with the DBH activities obtained in that particular group. Our results (11) on the developmental changes in Wistar rat serum aromatic L-amino acid decarboxylase (AADC) with L-DOPA and L-5-hydroxytryptophan as substrates and that of Kuzuya *et al*. (12) on DBH activity showed that the peak of AADC came at the age of 3- weeks, and for AADC again the peak activity appeared at the adult level. But the present developmental studies showed with Long-Evans rats that DBH activity came at 5 weeks of age and at no other developmental stages. This is very important to indicate the species variations in rats. This study was the first report on the developmental changes of DBH in various central and peripheral tissues, like, adrenal, liver, heart and brain tissues, like, brain stem, hypothalamus, caudate nucleus, colliculi, cerebral cortex, cerebellum, etc. Diets were provided with the same compositions to all the rats throughout the experimental periods and the concentration of ascorbic acid, Cu^2+^ and glucose were measured immediately after the tissues were collected. The results of these parameters were explainable with that of the DBH activities at various developmental stages (8-11). The results of the present studies showed that the developmental profiles of DBH enzyme in Long-Evans rats are quite different from that of other species of rats and mammals. and the results would be of great help to understand the nerve maturation and catecholeamines neurotransmitters formation in various mammals and humans.
